# Detection and Early Phase Assessment of Radiation-Induced Lung Injury in Mice Using Micro-CT

**DOI:** 10.1371/journal.pone.0045960

**Published:** 2012-09-24

**Authors:** Shigeyoshi Saito, Kenya Murase

**Affiliations:** Department of Medical Physics and Engineering, Division of Medical Technology and Science, Faculty of Health Science, Graduate School of Medicine, Osaka University, Osaka, Japan; University of Navarra, Spain

## Abstract

Radiation therapy is an important therapeutic modality for thoracic malignancies. However, radiation-induced pulmonary injuries such as radiation pneumonitis and fibrosis are major dose-limiting factors. Previous research shows that micro-computed tomography (micro-CT) can detect radiation-induced lung injuries a few months following irradiation, but studies to assess the early response of lung tissue are lacking. The aim of this study was to determine if micro-CT could be used to detect and assess early-phase radiation–induced lung injury in mice. Twenty-one animals were divided into three groups: normal (n = 7), one day after x-ray exposure (n = 7), and at four days after x-ray exposure (n = 7). The x-ray-exposed groups received a single dose of 20 Gy, to the whole lung. Histology showed enlargements of the air space (*Lm*: mean chord length) following irradiation. 40.5±3.8 µm and 60.0±6.9 µm were observed after one and four days, respectively, compared to 26.5±3.1 µm in normal mice. Three-dimensional micro-CT images were constructed and histograms of radiodensity - Hounsfield Units (HU) - were used to assess changes in mouse lungs. Radiation-induced lung injury was observed in irradiated mice, by the use of two parameters which were defined as shifts in peak HU between −200 to −800 HU (*Peak_HU_*) and increase in the number of pixels at −1000 HU (*Number_-1000_*). These parameters were correlated with histological changes. The results demonstrate that micro-CT can be used for the early detection and assessment of structural and histopathological changes resulting from radiation-induced lung injury in mice. Micro-CT has the advantage, over traditional histological techniques, of allowing longitudinal studies of lung disease progression and assessment of the entire lung, while reducing the number of animals required for such studies.

## Introduction

Radiation methods such as x-rays and charged particles are used in the treatment of patients with thoracic malignancies depending on the location, extent and pathologic diagnosis of the disease [1], [2]. Radiation therapy is one of the most important therapeutic modalities for thoracic malignancies. However, radiation-induced pulmonary injuries such as radiation pneumonitis and fibrosis are major dose-limiting factors when irradiating the thorax [3], [4]. Radiation pneumonitis usually occurs a few weeks, and radiation fibrosis occurs a few months after the completion of radiation therapy. Micro-computed tomography (Micro-CT) offers a non-invasive method for detecting and assessing radiation-induced lung injuries in thoracic radiotherapy patients.

Micro-CT refers to computed tomography conducted on a microscopic level and comparable to that achievable on a clinical CT system in human subjects [5]. It is currently widely used to evaluate bone specimens noninvasively [6] and it has also been applied to static vascular and lung imaging [7], [8]. *In-vivo* lung tumor studies using micro-CT have produced images in which lung nodules are detectable [9], and it is a novel tool for monitoring acute and chronic lung disease states in small animals [10], [11], [12]. Past research shows that the high-dose single radiation-induced lung injuries can be observed using micro-CT images a few months following irradiation [11], [13]. However, there are no studies which assess the early lung tissue response to high-dose single radiation exposure using micro-CT. Here we examined the potential of *in-vivo* micro-CT as a non-invasive tool to assess the progression of lung injury by radiation exposure in the first four days following x-ray irradiation.

The goal of the present study was to evaluate whether micro-CT could be used to detect and quantify early phase radiation-induced lung injury (one and four days after x-ray exposure), by comparing micro-CT results to parallel changes in lung histology. Coronal cross sections of mouse lungs, corresponding to micro-CT images, were stained with haematoxylin and eosin (H&E) and histological changes were compared to Hounsfield Unit (HU) assessments of three-dimensional micro-CT images. We show that micro-CT can be used for the detection and quantitative analysis of early structural and histopathological changes associated with radiation-induced lung injury in mice.

## Materials and Methods

### Animal Preparation

The Animal Welfare Committee of Osaka University approved this study. The mice used in this study, 8-week-old C57BL/6 males (n = 21, 23.2±1.5 g, Japan SLC, Hamamatsu, Japan) were allowed to rest for one week before the experiment. The animals had free access to food and water, and they were kept under standard laboratory conditions (a room temperature of 22–23°C, approximately 50% humidity, and a 12 h light/dark cycle). Twenty-one mice were divided into three groups: no-radiation controls (Control mice, n = 7), and two x-ray exposure groups, assessed one day (X1 mice; n = 7) or four days (X4 mice; n = 7) following exposure. The x-ray exposed groups received a single whole-lung x-ray consisting of a 20 Gy dose of radiation, previously shown to produce serious lung injury [14]. The x-rays were delivered at a dose rate of 0.88 Gy/min (120 kV, 15 mA with 1 mm thick aluminum filter, Rigaku Radioflex 350 X-ray generator), to a 2 cm×2 cm field and irradiated whole lung of each mouse. The rest of the body was shielded by 0.5 cm thick Pb. The radiation-exposed groups were kept under regular light/dark cycles until the micro-CT experiments were performed, either one or four days following x-ray exposure.

### Micro-CT

Computed tomography images were acquired using three-dimensional micro-CT (RmCT; Rigaku Co., Tokyo, Japan) with a resolution of 117×117×117 µm^3^, a tube voltage of 90 kV, and a tube current of 160 µA. Exposure time was 34 s and images were retrospectively gated at expiration breathing phase. During the CT scan, the mice were held in place by surgical tape and anesthetized through a facemask with 2% isoflurane. Tomographic images were obtained using 3D imaging software (i-VIEW; Morita Co., Tokyo, Japan). Three-dimensional reconstruction was performed using 512 of these images, processed by the volume rendering method, and HU (estimates of tissue density) were calculated and used to construct histograms of the lung. Areas of high density (between −200 to −500 HU) in three-dimensional Micro-CT slices were shown in green, and areas of low density (below −500 HU) were shown in blue. Two HU parameters, the peak HU of −200 to −800 HU (*Peak_HU_*) and the number of pixels at −1000 HU (*Number_-1000_*; air or empty space), were used to assess radiation-induced lung damage and differences among the three groups. The mice lung volumes were manually measured by the use of 3D micro-CT images using ImageJ software (Ver.1.40 g, National Institutes of Health, Bethesda, Maryland, USA).

### Histology

After CT scanning, all mice were sacrificed for histology, to determine the source of HU alterations in the lungs. Mice were euthanized by an overdose of pentobarbital (Dainippon Sumitomo Pharma Co., Ltd., Japan) and were prepared for histology by trans-cardiac perfusion with saline containing heparin followed by 7.5% paraformaldehyde. Extracted lungs were embedded in paraffin, and 4 µm coronal sections were cut, corresponding to the coronal diagram and orientations on the CT images. Slides were stained with haematoxylin and eosin (H&E) stain, to assess morphological alterations in lung tissue. Large nonalveolar structures such as blood vessels were removed from the image and the mean air space, chord length (*Lm*), was calculated for each section under light microscopy [15]. Each section produced five non-overlapping fields, and 10 sections were analyzed for each animal, resulting in 50 non-overlapping fields [16]. The observer manually drew a region of interest (ROI) on each slice that included the lung tissues without the blood vessel regions.

### Statistical Analysis

All statistical data are presented as mean ± standard deviation. Statistical analyses were performed using Prism5 (Prism, Version 5, USA) and were focused on the differences in HU parameters between the normal and radiation-exposed groups. Kruskal-Wallis test followed by Dunn's multiple comparison test was used to determine the significant differences in mean *Peak_HU_*, *Number_-1000_*, *Lm* and lung volume, among the treatment groups. Differences were considered significant if *P*<0.05. Correlation analyses were also conducted to determine correlations between micro-CT (*Peak_HU_* and *Number_-1000_*) and histological (air space, *Lm*) data.

## Results

### Animal Condition

The mean body weight of the mice one day after radiation-exposure (X1) was 25.6±0.9 g, similar to the mean body weight of the normal mice (25.4±1.3 g). No significant difference was observed between the control and X1 groups. In contrast, the body weights of the irradiated mice four days after exposure (X4) were 22.6±1.9 g. The body weights in the X4 mice were significantly decreased compared to those for the normal control mice (p<0.001).

The lung volume of the mice one day after radiation-exposure (X1) was 577±63 mm^3^, which was similar to the lung volume of the normal mice (582±52 mm^3^). The lung volume of the irradiated mice four days after exposure (X4) was 552±51 mm^3^. No significant difference was observed between the three groups.

### Micro-CT

**Figure 1:**
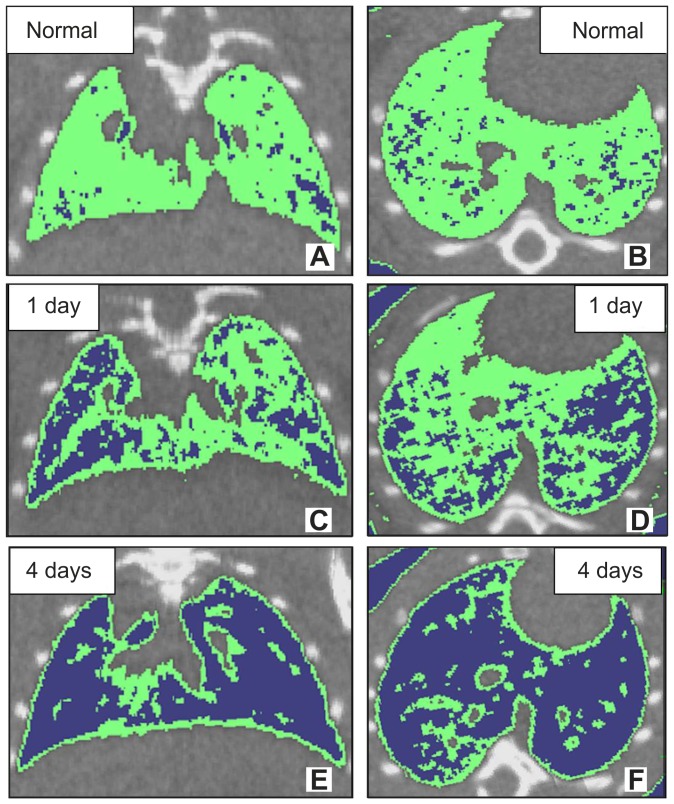
Micro-CT images. Typical images of micro-CT in normal (A and B) and radiation-induced lung injury mice (C, D, E and F). Top panel: micro-CT images of normal mouse lung (A and B). Middle panel: micro-CT images of radiation-induced mouse lung injury at 1 day after x-ray exposure (C and D). Bottom panel: micro-CT images of radiation-induced mouse lung injury at 4 days after X-ray exposure (E and F). (A, C and E) transaxial slice orientation. (B, D and F) horizontal slice orientation. Areas of density between −200 to −500 HU on three-dimensional micro-CT slices were shown in green. Areas of density below −500 HU on three-dimensional micro-CT slices are shown in blue.

Figure 1 shows images of representative micro-CT slices, in three-dimensional reconstructions, obtained from entire micro-CT data sets at three time points. Micro-CT images of normal mice lung are shown in Figure 1A and 1B, and of X1 and X4 mice in Figure 1C, 1D, 1E and 1F. Three-dimensional micro-CT slices show areas of density between −200 to −500 HU in green at different points (Figure 1). Horizontal micro-CT slices are shown in Figure 1A, 1C and 1E, while transaxial micro-CT slices are shown at different points (Figure 1B, 1D and 1F), and then the areas of density below −500 HU are blue (Figure 1).

**Figure 2:**
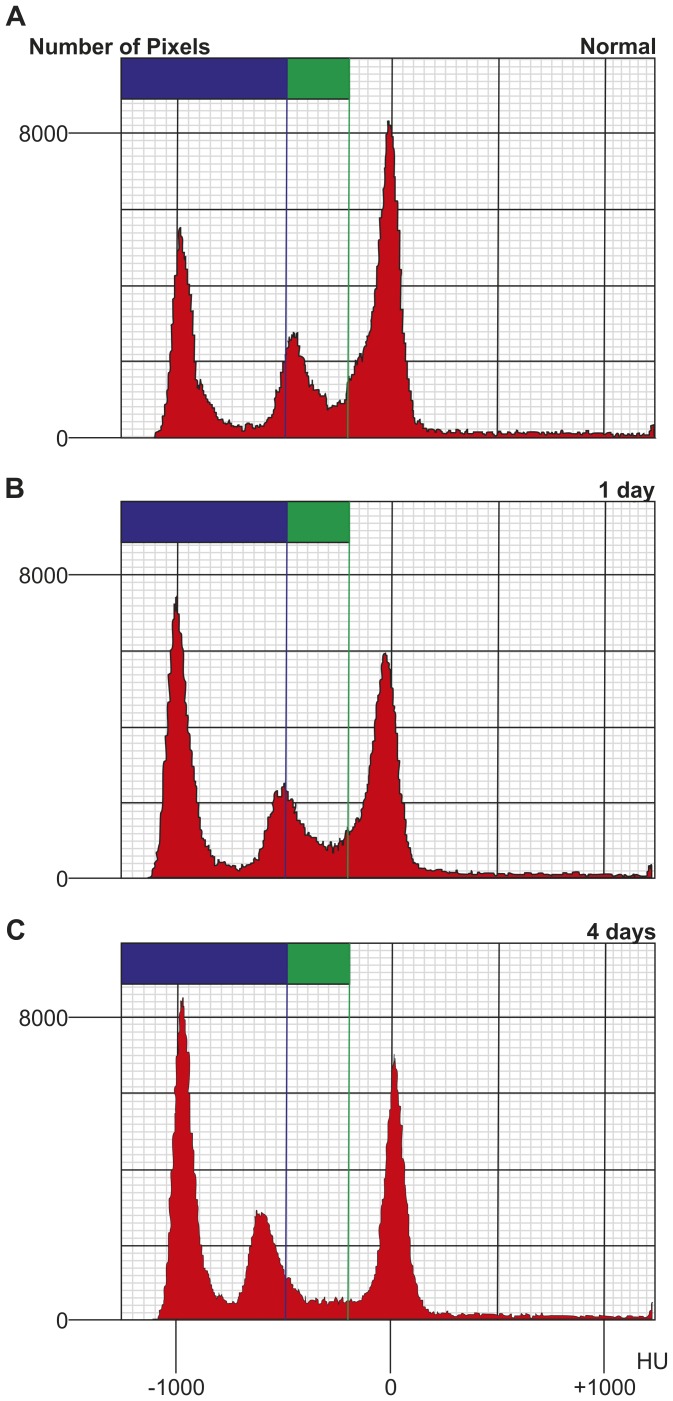
HU analysis. (A)Typical histograms of micro-CT in normal, (B) Radiation-induced mice lung injury at 1 day (X1), and (C) Radiation-induced mice lung injury at 4 days, after x-ray exposure (X4). Areas of high density (between −200 to −500 HU) in three-dimensional Micro-CT slices are shown in green, and areas of low density (below −500 HU) are shown in blue.

Figure 2 shows representative histograms of lungs from each group. Histograms were automatically calculated from the micro-CT images using 3D imaging software. Radiation-induced lung injury was observed as a shift in *Peak_HU_* and an increase in *Number_-1000_*. As can be seen from the early phase experiment (Figure 2B and 2C), the *Peak_HU_* values of radiation-treated X1 (−501.0±24.8 HU) were lower than the mean *Peak_HU_* of control animals (−456.4±8.9 HU). Especially, the *Peak_HU_* values of X4 animals (−615.4±10.9 HU, p<0.01) were significantly lower than the mean *Peak_HU_* of control animals. The *Number_-1000_* values at X1 (7303.8±245.8 pixels) following radiation exposure were greater than the *Number_-1000_* of normal mice lungs (5303.0±463.8 pixels). Moreover, the *Number_-1000_* values at X4 (8255.6±307.7 pixels, p<0.01) following radiation exposure were significantly greater than that of normal mice lungs.

### Histology

**Figure 3:**
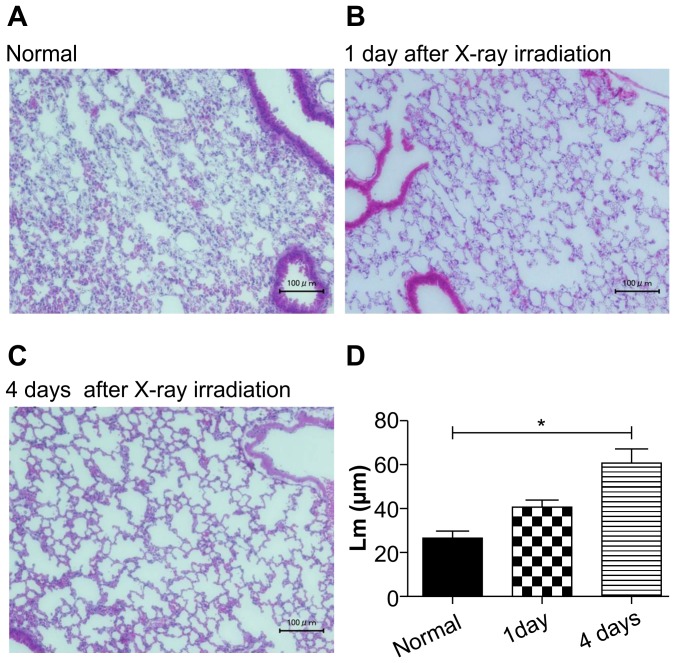
H & E stained histological sections of mouse lungs. Representative images of H & E stained sections of (A) normal; (B) X1, 1 day after radiation exposure; (C) X4, 4 days after radiation lungs. Scale bars represent 100 µm. (D) Comparison of mean air space (Lm values) between groups. The mean air spaces (Lm) in irradiated lungs, one and four days following irradiation, were greater than in control lungs. A significant increase was also observed between lungs four days following x-ray exposure (*; P<0.001).

H&E staining of coronal lung sections showed visible destruction of lung tissue in some mice at one and four days following irradiation, but the severity of lung damage varied between mice (Figure 3B and 3C). Enlargement of the air space (*Lm*) in radiation-injured lungs occurred at day 1 (40.5±3.8 µm) and showed a significant increase even at day 4 (60.0±6.9 µm, p<0.001) after x-ray exposure. In comparison, normal mice had a mean lung air space of 26.5±3.1 µm (Figure 3D).

### Correlation between Micro-CT and Histological Data

**Figure 4:**
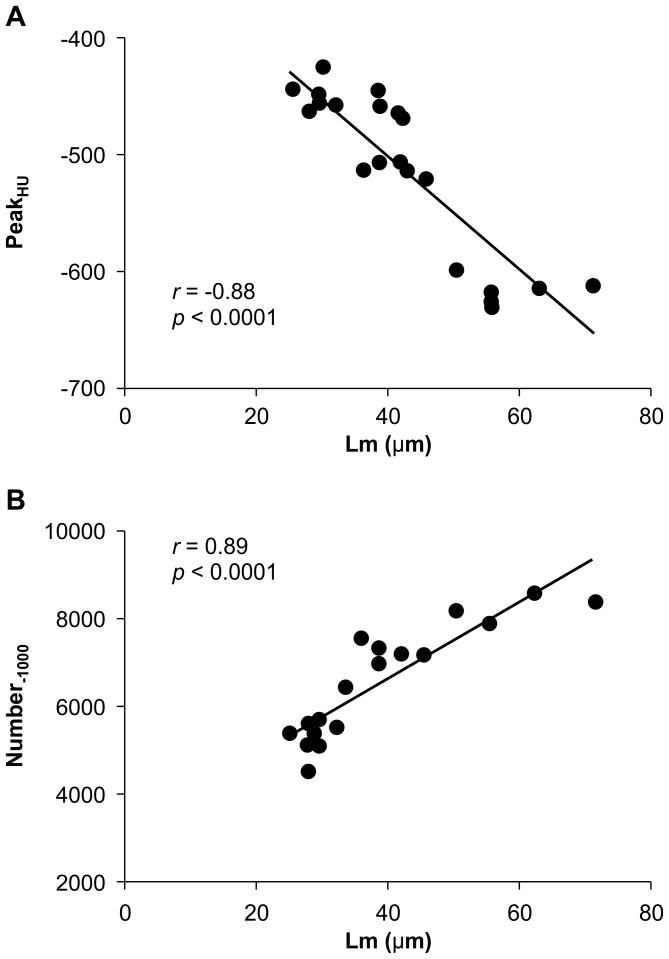
Correlation between micro-CT and histopathological data. (A) Correlation between *Peak_HU_* (position of the peak between −200 to −800 HU) and LM (air space, determined from H & E stained coronal lung sections); (B) Correlation between the number of pixels at −1000 HU (*Number_-1000_*) and Lm. Results of correlation analyses (r and *P* values) are shown.

Statistical correlations between micro-CT HU data, *Peak_HU_* and *Number_-1000_*, and histological data (*Lm*) were evaluated. A significant negative correlation was observed between *Peak_HU_* and *Lm* (r = −0.88, P<0.0001, Figure 4A) and a positive correlation was existed between *Number_-1000_* and *Lm* (r = 0.89, P<0.0001, Figure 4B).

## Discussion

Histological analysis is the most common technique used to quantify the extent of lung injury in rodent models [17]. However, correlation between micro-CT images and histological changes has been shown in emphysema mice [12], [18], [19], [20]. The present study shows that micro-CT can also be used for the detection and quantitative analysis of early (one and four days after x-ray exposure) structural and histopathological changes associated with radiation-induced lung injury. HU analysis of three-dimensional micro-CT images showed that two HU parameters, *Peak_HU_* and the *Number_-1000_*_,_ were highly correlated with air space enlargement (*Lm*) in radiation-exposed mice. The present study shows that micro-CT imaging of radiation-induced pulmonary injury is not only able to provide reliable quantification of air space enlargement (Figure 3B and 3C) but also has several advantages over the conventional, histological methods.

Radiation injury of the lung is classified into early and late phase [21]. The early phase injury consists of cellular infiltration that is predominantly composed of macrophages and appears as ground-glass opacities in radiological experiments [22], [23]. Radiation pneumonitis is most prominent at 3–4 months after radiotherapy. The late phase lung injury radiation fibrosis is marked by collagen deposition and fibrosis and appears as volume loss, consolidation, and traction bronchiectasis in radiological experiments, [22], [23], [24] Consistent with the classical phases of radiation-induced lung injury, we did not observe volume loss and consolidation in our radiation-induced pulmonary injury model mice (Figure 3). However, we did observe a very early (at one and four days) response of the lung to radiation exposure (Figure 1E and 1F) detectable as HU alterations on three-dimensional CT images.

The late effect of radiation on the lungs can be usually divided into two syndromes, radiation pneumonitis, which develops within 6 months after X - rays exposure below 10 Gy and radiation fibrosis which is a delayed or late reaction that develops from about 6 months to years after 10–15 Gy exposure [25]. The previous studies have revealed that the time course of deaths in mice following thoracic irradiation is considerably prolonged [26]. The irradiated dose that resulted in 50% mortality was around 15 Gy, similar to the value found by Phillips et al. [27] and Field et al. [28]. We can observe very early response to radiation over 15 Gy (20 Gy) using micro-CT study. Maisin [29] reported the use of electron microscopy for the evaluation of lung damage very early post-irradiation. After a dose of 20 Gy to the rat lung, he observed focal lesions involving changes in cell ultrastructure within 3 hours of x-ray irradiation. These alterations continued, and by 6 hours, cellular ultrastructure was markedly altered. Adamson et al. described similar endothelial changes in rat lung within 2 days of 11 Gy and 5 days of 6.5 Gy [30]. Moreover, it has been reported that the initial site of damage was the capillaries and their endothelium one-side of lung irradiation with x-rays [31]. These alterations were minimal in the 2–8 Gy group, but most significant within the highest dose one (24–32 Gy). At very high doses (over 10 Gy), radiation can cause cessation of metabolism and cellular disintegration, a type of cell death known as interphase death. However, much lower doses (2–3 Gy) will inhibit cell division. Moreover, the high-dose effect is due to direct radiation induced cell-death, while low dose response may imply the intervention of additional mechanisms such as early local inflammation. A Hounsfield Unit assessment was performed on three-dimensional micro-CT reconstructions and this data used to construct histograms of the lung tissue density. The response to radiation was observed as a shift in the *Peak_HU_* and increase in *Number-_1000_* (Figure 2). Based on our histological results, it appears as though radiation exposure causes a severe alveolar wall breakdown one and four days after x-ray irradiation (Figures 3B and 3C). Enlargement of the air spaces (*Lm*) in radiation injury lung was significant at day 1, and increase further at day 4, after x-ray exposure. These results contrast with the findings of Downing et al. [32] who, albeit at lower radiation doses, showed increases in septal thickness at 24 hours. Previous reports indicate that various inflammatory mediators or cytokines released from injured alveolar cells produce inflammation in the same time frame [33], [34], [35]. Furthermore, the alveolar space is filled with exudates resulting from direct radiation injury and inflammatory processes. This suggests that the enlarged alveolar space, caused by filling with exudates and expansion, was responsible for the HU (*Number_-1000_*) increase in radiation-exposed lungs. However, we observed a very early response of the lung to radiation exposure, as HU (*Peak_HU_)* decreases on micro-CT images. While the relevance of this very early and transient phase remains controversial and may be a function of different mouse strains [11], our results demonstrate that three-dimensional micro-CT has the potential to detect and quantify very early responses of the whole lung to radiation injury and offers several advantages over the conventional methods.

First, noninvasive CT scanning does not require euthanasia and can be performed as needed at several time points using the same animals. Also, the radiation dose of typical micro-CT scans used for mouse lungs is below 10 to 30 mGy [35], [36], [37], [38], over 50 times less than the values capable of inducing significant lung damage [25]. Therefore, it can thus be repeated in individual animals, i.e., micro-CT enables longitudinal studies. Here we show that micro-CT can be used to quantify early phase changes associated with radiation-induced lung injury and previous research demonstrated its value in later stages [11], [13]. Therefore, micro-CT offers the opportunity to visualize dynamic disease progression.

Moreover, three-dimensional information can be quickly obtained from micro-CT using simple image analysis tools. The retrospectively respiratory-gated micro-CT imaging technique can provide three-dimensional images from a single 34-second scan. The data can be then reconstructed in 1 minute and further analyzed for three-dimensional assessment using animal imaging software in another few minutes. Conventional research into lung disease using animal models relies on mean chord length measurements taken from random areas of the lung and then averaged [15], [17]. This analysis is usually performed on a single or few sections of lung, thereby missing potential patchy areas of air space enlargement. Similar limitations may apply to two-dimensional CT imaging. In contrast, three-dimensional micro-CT images can provide information on the spatial distribution and heterogeneity of the lung injury. These attributes of micro-CT not only allow the investigator to account for inter-individual variability but also reduce the number of animals required for a study.

While this study is the first to demonstrate the use of micro-CT for the detection and study of early phase radiation-induced lung injury in mice, it was not without limitations. A major limitation is that this study covers only the first few days following irradiation of the lung, and it has yet to be established how these changes actually relate to lung injury that develops a few months later. Also, there was a considerable change in the extent of injury between one and four days after irradiation. An analysis of the biological events taking place during this time would have yielded extra information on each technique, and provided a better understanding of disease progression. However, we chose to investigate lung injury at these time points in order to assess the sensitivity of micro-CT. In this study, the evaluation of radiation lung injury can be seen as a shift in the *Peak_HU_* and an increase in the *Number_-1000_*. These values are similar to other thresholds (around −600 HU) previously used by other investigators for assessment of emphysema model mice [18], [20]. The measurement of Hounsfield Units by the use of micro-CT was not consistent, with some pixels values being under −1000 (Fig. 2). Further studies, including comparisons of micro-CT with macro- and microscopic changes in morphometry, are warranted to establish the most appropriate cut-off value and correction the precise HU values for estimating the extent of lung injury.

In conclusion, both micro-CT and histomorphometry give reliable measurements of air space enlargement, associated with early (one and four days after irradiation) phase radiation-induced lung injury in mice. Micro-CT offers advantages, by enabling longitudinal studies and providing a three-dimensional analysis of the entire lung, thus reducing the required number of animals required for studies and providing more relevant information on the progression of lung disease.

## References

[1] Okada T, Kamada T, Tsuji H, Mizoe JE, Baba M, et al (2010) Carbon ion radiotherapy: clinical experiences at National Institute of Radiological Science (NIRS). Journal of radiation research 51: 355–364. 20508375 10.1269/jrr.10016

[2] Grutters JP, Kessels AG, Pijls-Johannesma M, De Ruysscher D, Joore MA, et al (2010) Comparison of the effectiveness of radiotherapy with photons, protons and carbon-ions for non-small cell lung cancer: a meta-analysis. Radiotherapy and oncology : journal of the European Society for Therapeutic Radiology and Oncology 95: 32–40. 19733410 10.1016/j.radonc.2009.08.003

[3] Toma CL, Ciprut T, Bugarin S, Rosca D, Bogdan MA (2011) [Radiation induced lung injuries secondary to radiotherapy for breast cancer]. Pneumologia 60: 40–46. 21548199

[4] Ding X, Ji W, Li J, Zhang X, Wang L (2011) Radiation recall pneumonitis induced by chemotherapy after thoracic radiotherapy for lung cancer. Radiation oncology 6: 24. 21375774 10.1186/1748-717X-6-24 PMC3063220

[5] Paulus MJ, Gleason SS, Kennel SJ, Hunsicker PR, Johnson DK (2000) High resolution X-ray computed tomography: an emerging tool for small animal cancer research. Neoplasia 2: 62–70. 10933069 10.1038/sj.neo.7900069 PMC1531867

[6] Yamashita T, Nabeshima Y, Noda M (2000) High-resolution micro-computed tomography analyses of the abnormal trabecular bone structures in klotho gene mutant mice. The Journal of endocrinology 164: 239–245. 10657859 10.1677/joe.0.1640239

[7] Jorgensen SM, Demirkaya O, Ritman EL (1998) Three-dimensional imaging of vasculature and parenchyma in intact rodent organs with X-ray micro-CT. The American journal of physiology 275: H1103–1114. 9724319 10.1152/ajpheart.1998.275.3.H1103

[8] Nakaya Y, Omatsu H, Matsui E, Niki N, Moriyama N (2002) [Development of Micro CT and it's usefulness for structural analysis of pulmonary disease]. Nihon Hoshasen Gijutsu Gakkai zasshi 58: 885–892. 12515953 10.6009/jjrt.kj00001364326

[9] Kennel SJ, Davis IA, Branning J, Pan H, Kabalka GW, et al (2000) High resolution computed tomography and MRI for monitoring lung tumor growth in mice undergoing radioimmunotherapy: correlation with histology. Medical physics 27: 1101–1107. 10841415 10.1118/1.598974

[10] Shofer S, Badea C, Qi Y, Potts E, Foster WM, et al (2008) A micro-CT analysis of murine lung recruitment in bleomycin-induced lung injury. Journal of applied physiology 105: 669–677. 18566189 10.1152/japplphysiol.00980.2007 PMC2519942

[11] Jackson IL, Vujaskovic Z, Down JD (2010) Revisiting strain-related differences in radiation sensitivity of the mouse lung: recognizing and avoiding the confounding effects of pleural effusions. Radiation research 173: 10–20. 20041755 10.1667/RR1911.1 PMC2818983

[12] Artaechevarria X, Blanco D, de Biurrun G, Ceresa M, Perez-Martin D, et al (2011) Evaluation of micro-CT for emphysema assessment in mice: comparison with non-radiological techniques. European radiology 21: 954–962. 20953986 10.1007/s00330-010-1982-5

[13] Jackson IL, Vujaskovic Z, Down JD (2011) A further comparison of pathologies after thoracic irradiation among different mouse strains: finding the best preclinical model for evaluating therapies directed against radiation-induced lung damage. Radiation research 175: 510–518. 21338245 10.1667/RR2421.1 PMC3110676

[14] Abdollahi A, Li M, Ping G, Plathow C, Domhan S, et al (2005) Inhibition of platelet-derived growth factor signaling attenuates pulmonary fibrosis. The Journal of experimental medicine 201: 925–935. 15781583 10.1084/jem.20041393 PMC2213091

[15] Mitzner W (2008) Use of mean airspace chord length to assess emphysema. Journal of applied physiology 105: 1980–1981. 18719230 10.1152/japplphysiol.90968.2008 PMC4587590

[16] Hind M, Maden M (2004) Retinoic acid induces alveolar regeneration in the adult mouse lung. The European respiratory journal : official journal of the European Society for Clinical Respiratory Physiology 23: 20–27. 10.1183/09031936.03.00119103 14738226

[17] Parameswaran H, Majumdar A, Ito S, Alencar AM, Suki B (2006) Quantitative characterization of airspace enlargement in emphysema. Journal of applied physiology 100: 186–193. 16166240 10.1152/japplphysiol.00424.2005

[18] Postnov AA, Meurrens K, Weiler H, Van Dyck D, Xu H, et al (2005) In vivo assessment of emphysema in mice by high resolution X-ray microtomography. Journal of microscopy 220: 70–75. 16269065 10.1111/j.1365-2818.2005.01510.x

[19] Ford NL, Martin EL, Lewis JF, Veldhuizen RA, Holdsworth DW, et al (2009) Quantifying lung morphology with respiratory-gated micro-CT in a murine model of emphysema. Physics in medicine and biology 54: 2121–2130. 19287083 10.1088/0031-9155/54/7/018

[20] Froese AR, Ask K, Labiris R, Farncombe T, Warburton D, et al (2007) Three-dimensional computed tomography imaging in an animal model of emphysema. The European respiratory journal : official journal of the European Society for Clinical Respiratory Physiology 30: 1082–1089. 10.1183/09031936.00000507 17804451

[21] Nicholas D, Down JD (1985) The assessment of early and late radiation injury to the mouse lung using X-ray computerised tomography. Radiotherapy and oncology : journal of the European Society for Therapeutic Radiology and Oncology 4: 253–263. 4081113 10.1016/s0167-8140(85)80090-6

[22] Park HJ, Kim KJ, Park SH, Kay CS, Oh JS (2009) Early CT findings of tomotherapy-induced radiation pneumonitis after treatment of lung malignancy. AJR American journal of roentgenology 193: W209–213. 19696261 10.2214/AJR.08.2298

[23] Choi YW, Munden RF, Erasmus JJ, Park KJ, Chung WK, et al.. (2004) Effects of radiation therapy on the lung: radiologic appearances and differential diagnosis. Radiographics : a review publication of the Radiological Society of North America, Inc 24: 985–997; discussion 998. 10.1148/rg.244035160 15256622

[24] Kimura T, Matsuura K, Murakami Y, Hashimoto Y, Kenjo M, et al (2006) CT appearance of radiation injury of the lung and clinical symptoms after stereotactic body radiation therapy (SBRT) for lung cancers: are patients with pulmonary emphysema also candidates for SBRT for lung cancers? International journal of radiation oncology, biology, physics 66: 483–491. 10.1016/j.ijrobp.2006.05.008 16904838

[25] Coggle JE, Lambert BE, Moores SR (1986) Radiation effects in the lung. Environmental health perspectives 70: 261–291. 3549278 10.1289/ehp.8670261 PMC1474274

[26] Steel GG, Adams K, Peckham MJ (1979) Lung damage in C57B1 mice following thoracic irradiation: enhancement by chemotherapy. The British journal of radiology 52: 741–747. 476389 10.1259/0007-1285-52-621-741

[27] Phillips TL, Wharam MD, Margolis LW (1975) Modification of radiation injury to normal tissues by chemotherapeutic agents. Cancer 35: 1678–1684. 50122 10.1002/1097-0142(197506)35:6<1678::aid-cncr2820350629>3.0.co;2-k

[28] Field SB, Hornsey S, Kutsutani Y (1976) Effects of fractionated irradiation on mouse lung and a phenomenon of slow repair. The British journal of radiology 49: 700–707. 953389 10.1259/0007-1285-49-584-700

[29] Maisin JR (1970) The ultrastructure of the lung of mice exposed to a supra-lethal dose of ionizing radiation on the thorax. Radiation research 44: 545–564. 5490503

[30] Adamson IY, Bowden DH, Wyatt JP (1970) A pathway to pulmonary fibrosis: an ultrastructural study of mouse and rat following radiation to the whole body and hemithorax. The American journal of pathology 58: 481–498. 5436095 PMC2032842

[31] Moosavi H, McDonald S, Rubin P, Cooper R, Stuard ID, et al (1977) Early radiation dose-response in lung: an ultrastructural study. International journal of radiation oncology, biology, physics 2: 921–931. 10.1016/0360-3016(77)90190-0 591409

[32] Downing L, Sawarynski KE, Li J, McGonagle M, Sims MD, et al (2010) A simple quantitative method for assessing pulmonary damage after x irradiation. Radiation research 173: 536–544. 20334526 10.1667/RR1712.1

[33] Rubin P, Johnston CJ, Williams JP, McDonald S, Finkelstein JN (1995) A perpetual cascade of cytokines postirradiation leads to pulmonary fibrosis. International journal of radiation oncology, biology, physics 33: 99–109. 10.1016/0360-3016(95)00095-G 7642437

[34] Ibuki Y, Goto R (1997) Enhancement of NO production from resident peritoneal macrophages by in vitro gamma-irradiation and its relationship to reactive oxygen intermediates. Free radical biology & medicine 22: 1029–1035. 9034242 10.1016/s0891-5849(96)00500-x

[35] Chen Y, Williams J, Ding I, Hernady E, Liu W, et al (2002) Radiation pneumonitis and early circulatory cytokine markers. Seminars in radiation oncology 12: 26–33. 11917281 10.1053/srao.2002.31360

[36] Kameoka S, Matsumoto K, Kai Y, Yonehara Y, Arai Y, et al (2010) Establishment of temporomandibular joint puncture technique in rats using in vivo micro-computed tomography (R_mCT(R)). Dento maxillo facial radiology 39: 441–445. 20841463 10.1259/dmfr/37174063 PMC3520191

[37] Figueroa SD, Winkelmann CT, Miller HW, Volkert WA, Hoffman TJ (2008) TLD assessment of mouse dosimetry during microCT imaging. Medical physics 35: 3866–3874. 18841837 10.1118/1.2959847 PMC2809703

[38] Rodt T, Luepke M, Boehm C, von Falck C, Stamm G, et al (2011) Phantom and cadaver measurements of dose and dose distribution in micro-CT of the chest in mice. Acta radiologica 52: 75–80. 21498330 10.1258/ar.2010.100059

